# Development and validation of a deep learning model for predicting postoperative survival of patients with gastric cancer

**DOI:** 10.1186/s12889-024-18221-6

**Published:** 2024-03-06

**Authors:** Mengjie Wu, Xiaofan Yang, Yuxi Liu, Feng Han, Xi Li, Jufeng Wang, Dandan Guo, Xiance Tang, Lu Lin, Changpeng Liu

**Affiliations:** 1 Department of Medical Oncology, Affiliated Cancer Hospital of Zhengzhou University, Henan Cancer Hospital, Zhengzhou, 450008 China; 2 Department of Medical Records, Office for DRGs (Diagnosis Related Groups), Affiliated Cancer Hospital of Zhengzhou University, Henan Cancer Hospital, No. 127 Dongming Rd, PO Box 0061, Zhengzhou, Henan Province 450008 China; 3 Department of Radiology, The Third Affiliated Hospital of Zhengzhou University, Zhengzhou, China; 4 Translational Medicine Research Center, People’s Hospital of Henan University of Chinese Medicine, Zhengzhou People’s Hospital, Zhengzhou, Henan 450003 China

**Keywords:** Machine learning, Deep learning, Gastric cancer, Predictive model, Survival rate

## Abstract

**Background:**

Deep learning (DL), a specialized form of machine learning (ML), is valuable for forecasting survival in various diseases. Its clinical applicability in real-world patients with gastric cancer (GC) has yet to be extensively validated.

**Methods:**

A combined cohort of 11,414 GC patients from the Surveillance, Epidemiology and End Results (SEER) database and 2,846 patients from a Chinese dataset were utilized. The internal validation of different algorithms, including DL model, traditional ML models, and American Joint Committee on Cancer (AJCC) stage model, was conducted by training and testing sets on the SEER database, followed by external validation on the Chinese dataset. The performance of the algorithms was assessed using the area under the receiver operating characteristic curve, decision curve, and calibration curve.

**Results:**

DL model demonstrated superior performance in terms of the area under the curve (AUC) at 1, 3, and, 5 years post-surgery across both datasets, surpassing other ML models and AJCC stage model, with AUCs of 0.77, 0.80, and 0.82 in the SEER dataset and 0.77, 0.76, and 0.75 in the Chinese dataset, respectively. Furthermore, decision curve analysis revealed that the DL model yielded greater net gains at 3 years than other ML models and AJCC stage model, and calibration plots at 3 years indicated a favorable level of consistency between the ML and actual observations during external validation.

**Conclusions:**

DL-based model was established to accurately predict the survival rate of postoperative patients with GC.

**Supplementary Information:**

The online version contains supplementary material available at 10.1186/s12889-024-18221-6.

## Background

Gastric cancer (GC) is one of the common malignant tumors, and surgical resection is still the only option to cure early GC and the main treatment for GC [1]. Even if patients with GC have undergone radical surgery, there are still many factors affecting their survival and disease progression, including clinical as well as pathological factors, such as stage, histological type, depth of infiltration and lymph node and distant metastasis [2–5]. Therefore, accurate prediction of postoperative survival rate is crucial for both patients and healthcare institutions. GC is a heterogeneous, multifactorial disease, and the variability of these multiple factors and the complexity of GC make treatment and survival prediction extremely difficult [6]. Currently, clinicians usually assess survival based on the American Joint Committee on Cancer (AJCC) stage combined with their own medical experience, overlooking the role of other survival-influencing factors [7]. The staging system is widely used and effective in guiding treatment decisions for GC. However, it fails to consider various factors such as sex, age, tumor size, and histopathological type, all of which can significantly influence survival prognosis. Additionally, traditional methods, such as Cox regression, in survival analysis encounter limitations including the requirement for proportional hazards and the assumption of linearity in continuous variables. These constraints may restrict their applicability in complex scenarios. However, deep learning-based prognostic models represent a significant advancement as they effectively address these issues. They can handle non-proportional hazards and model non-linear relationships between variables and outcomes, rendering them more versatile and accurate for survival prediction across diverse clinical settings.

Machine learning (ML) excels in acquiring information from high-dimensional, complex data, learning automatically and making predictions in supervised or unsupervised mode, and plays a major role in disease prognosis [8]. Compared to the AJCC stage model, ML predictive models may be more appropriate for clinical settings to guide clinical decision making. To the best of our knowledge, there is a lack of effective predictive models for the correlation of several clinical factors with the prognosis of GC patients after surgery. Deep learning (DL), a special kind of ML model that includes multiple neural networks, can handle more complex information. The DL method, compared to traditional ML models including the multitask logistic regression and random forest models, has many advantages. First, DL can learn complex patterns and representations from large datasets, resulting in superior performance compared to traditional ML algorithms. Second, DL algorithm can scale effectively with large amounts of data. Third, DL model has the ability to automatically learn and extract relevant features from raw data. At last, DL models can leverage pre-trained models on large datasets, allowing for transfer learning. This approach enables the application of existing knowledge from one domain to another, even with limited labeled data, thereby reducing the need for extensive training data. Some studies have utilized DL models for analysis in surgical oncology research [9–11]. However, most of these studies have focused on diagnostic applications, such as automated quantification of radiographic images, digital histopathologic image interpretation, or biomarker analysis [12–15]. To our knowledge, there are limited published studies utilizing DL models for prognostic prediction in surgical oncology, particularly in the field of GC. Therefore, DL-based survival analysis offers insights into survival prediction after GC surgery.

The Surveillance, Epidemiology and End Results (SEER) database, established by the National Cancer Institute, is a comprehensive cancer registry with well-developed and regularly updated data that provides a wealth of information on patient clinical characteristics, treatment, and survival data [16]. This study aims to extract information about postoperative GC patients from the SEER database, construct a GC survival prediction model by DL algorithm, and evaluate the accuracy of the constructed model using information collected from real-world GC patients to analyze the factors influencing the probability of GC survival and the 5-year survival status to provide decision support for clinical treatment and prognosis of GC.

## Methods

### Data collection and patient characteristics

The current research is a retrospective study that relies on data from the SEER database, which is maintained by the National Cancer Institute. The SEER database collects information on cancer incidence and survival rates from 18 cancer registries, covering approximately 27.8% of the US population. This study also includes patients diagnosed with gastric cancer between 2016 and 2020 at the Affiliated Cancer Hospital of Zhengzhou University, Henan Cancer Hospital, forming a Chinese dataset. All procedures involving human participants in this study followed the ethical standards established by the institutional and/or national research committee, as well as the 1964 Helsinki declaration and its subsequent amendments or equivalent ethical standards. Due to the retrospective nature of the study, informed consent from patients was not required.

In this study, individuals who were diagnosed with primary GC and met the following criteria were included: being above 18 years of age and having undergone surgical intervention. The identification of eligible patients was based on the definition of the primary tumor location, which encompassed the following categories: C16.0-Cardia, C16.1-Fundus of stomach; C16.2-Body of stomach; C16.3-Gastric antrum; C16.4-Pylorus; C16.5-Lesser curvature of stomach; C16.6-Greater curvature of stomach; C16.8-Overlapping lesion of stomach; C16.9-Stomach. Individuals with unknown age or survival duration were excluded from the study. For the purpose of analysis, important patient attributes were collected, which included the following information: age at diagnosis (in years), sex, tumor location, histology, grade, AJCC stage, tumor size (in millimeters), the number of examined lymph nodes (LN), the number of positive LNs, radiation treatment, and chemotherapy.

### Selection of proper ML algorithms

#### Multi-task logistic regression (MTLR)

The MTLR model presents a novel approach to survival analysis, extending traditional methods by directly modeling the survival function across multiple time intervals. The model accommodates the time-varying effects of covariates, allowing for a more nuanced understanding of risk factors. MTLR captures these dynamics, offering several advantages over Cox’s proportional hazards and Aalen’s additive models, which are traditional staples of survival analysis. Unlike these models, MTLR can handle non-proportional hazards and non-linear effects, resulting in improved predictive accuracy and flexibility.

A simplified view of the mathematical formulation behind MTLR is shown below:

Assume we divide the survival time into *N* discrete intervals, $[{t_0},{t_1}),[{t_1},{t_2}), \ldots,[{t_{N - 1}},{t_N}]$, where ${t_0}=0$ and ${t_N}=\infty$ (or some maximum follow-up time). For each interval *i*, MTLR models the probability that the event of interest (e.g., death) occurs within that interval, given that it has not occurred before ${t_i}$.

The probability of the event occurring in the *i*^th^ interval, given covariates X, is modeled as:

$$P(T \in [{t_{i - 1}},{t_i})|X)=\frac{{\exp (\beta _{i}^{T}X)}}{{\sum\limits_{{j=0}}^{N} {\exp } (\beta _{j}^{T}X)}}$$

where:

- *T* is the survival time,
- *X* represents the covariates (or features) of the patient,
- *βi* is the coefficient vector for interval *i*,
- *N* is the number of intervals.

Random Survival Forests (RSF) represent another innovative extension of Random Forests specifically adapted for survival analysis. RSF does not assume a specific form for the underlying hazard function, making it adaptable for various datasets. By aggregating predictions from multiple decision trees built on various subsamples of the data, RSF enhances prediction accuracy and robustness.

For a new observation, the survival function is estimated by aggregating predictions from all the trees in the forest.

In mathematical terms, if we denote *S*_*i*​_(*t*) as the survival function estimated by the *i*^th^ tree in the forest for time *t*, and *N* as the total number of trees, the overall survival function *S*(*t*) for an observation is given by:

$$S(t)=\frac{1}{N}\sum\limits_{{i=1}}^{N} {{S_i}} (t)$$

The DeepSurv model in PySurvival is a deep learning-based approach to survival analysis, renowned for its ability to capture complex, non-linear relationships in the data. DeepSurv extends traditional survival analysis models by using neural networks, allowing for more flexible and potentially more accurate modeling of survival data, especially when dealing with high-dimensional and complex datasets.

DeepSurv utilizes a neural network to model the hazard function. The hazard function in the context of DeepSurv can be expressed as:

$$h(t|X)\, = \,{h_0}(t)exp(g(X,\theta ))$$

where:

- $h(t|X)$ is the hazard function at time *t* given covariates *X*.
- ${h_0}(t)$ is the baseline hazard function, which is typically left unspecified.
- $exp(g(X,\theta ))$is a non-linear function represented by the neural network, with *X* being the input covariates and *θ* representing the network’s parameters.
- The neural network *g*(*X*,*θ*) learns complex relationships between the input covariates and the log-risk of the event occurring.

Our decision to employ DL, MTLR, and RF into our study, alongside a comparison with the traditional TNM staging system, stemmed from our aim to explore a spectrum of machine learning approaches that address the unique challenges posed by survival data. Each method was selected based on its specific strengths in addressing different aspects of survival analysis:

- DL was chosen for its unparalleled capability in modeling complex, non-linear relationships within high-dimensional datasets.
- MTLR was employed for its innovative approach to capturing time-varying effects of covariates across multiple time intervals.
- RSF was utilized for its effectiveness in handling censored data and reducing overfitting, leveraging the ensemble strength of Random Forests tailored to survival analysis.

Various ML algorithms were employed in this study. We implemented a two-stage validation process for our survival analysis models. Initially, we conducted internal validation using the SEER database, where the data was randomly split into two portions: 60% for model training and 40% for validation. This partitioning enabled us to develop and subsequently evaluate the model within the same dataset. We employed a grid search approach combined with the C-index to select parameters for our survival analysis model. This method entails exploring a predefined set of parameter combinations, training the model on a training dataset, and then evaluating its performance on internal validation dataset using the C-index. This process systematically identifies the optimal parameters that enhance the model’s predictive accuracy by ranking survival times effectively. The results of the grid search are provided in the supplementary materials. For external validation, we utilized an independent dataset from China, allowing us to assess the model’s performance in a different patient population. This comprehensive approach ensured a thorough evaluation of our models across diverse clinical contexts [17]. The ML algorithms that were tested in this study encompassed DL, MTLR and RF. The accuracy of these ML models was compared with TNM stage. In order to evaluate the performance of the model, various metrics were computed, including the area under the receiver operating characteristic curve [18]. Area Under the Curve (AUC) is a performance measure that remains unaffected by specific thresholds and provides a comprehensive evaluation of the model’s performance.

The AUC values range from 0.5 to 1.0, where 0.5 represents random chance and 1.0 represents perfect classification. Additionally, the calibration of the model, which compares predicted outcomes to observed outcomes, was evaluated through visual examination of calibration plots. Decision curve analysis was performed to calculate the clinical net benefit for each prediction model. The net benefit measures the advantages gained by using the model’s predictions to guide decision-making. The net benefit of employing these strategies was compared to the models that rely on prognosis-based interventions, meaning interventions based on a predicted risk exceeding a specific threshold.

### Statistical analysis

Categorical variables were compared for differences using the chi-square test, while the non-parametric Mann–Whitney test was used to compare differences between continuous variables. Univariate survival analysis was conducted using the Kaplan–Meier method, and the log-rank test was employed to compare the survival rates among different subgroups. The ML algorithms were trained and tested using Pysurvival, while SAS 9.4 was used for conducting survival analysis, chi-square tests, and Mann–Whitney tests. A significance level of *P* < 0.05 was considered statistically significant, using a two-sided test.

## Results

### Study population and baseline characteristics

The number of identified patients was 2,846 in the Chinese and 11,414 in the SEER registry dataset, with median ages at diagnosis of 60.8 and 65.3 years, respectively (60.75 ± 10.07 vs. 65.32 ± 13.58 years, *P* < 0.01). The proportion of male patients in the Chinese dataset was higher than that in the SEER dataset (75.54% vs. 62.18%, *P* < 0.01). Patients in the Chinese dataset were more likely to have tumors located in overlapping locations (24.49% vs. 7.23%, *P* < 0.01). However, patients in the SEER dataset were more likely to have tumors located in the gastric antrum (21.34% vs. 15.39%, *P* < 0.01). Most patients in the Chinese dataset had grade II (55.48%) and T4 (51.65%), while patients in the SEER dataset had grade III and IV (60.55%) and T3 (36.00%). Patients in the Chinese dataset received less radiation (3.62% vs. 34.33%) and more chemotherapy (75.38% vs. 55.68%) than those in the SEER dataset. Among 11 variables included in this study, all parameters showed significant difference between the SEER and Chinese datasets (Table 1).

**Table 1:** Characteristics of included patients in Chinese and SEER dataset

	Chinese dataset (*n* = 2846)	SEER dataset (*n* = 11,414)	p-value
**Years of diagnosis**	2016–2020	2010–2015	
**Last follow up date**	2023	2020	
**Age**	60.75 ± 10.07	65.32 ± 13.58	*P* < 0.01
**Sex**			*P* < 0.01
Male	2150(75.54)	7097(62.18)	
Female	696(24.46)	4317(37.82)	
**Tumor location** ^**a)**^			*P* < 0.01
Cardia	849(29.83)	3493(30.6)	
Fundus	17(0.60)	492(4.31)	
Body	536(18.83)	1206(10.57)	
Gastric antrum	438(15.39)	2436(21.34)	
Pylorus	29(1.02)	337(2.95)	
Lesser curvature	101(3.55)	1158(10.15)	
Greater curvature	14(0.49)	580(5.08)	
Overlapping	697(24.49)	825(7.23)	
Stomach	165(5.80)	887(7.77)	
**Histology** ^**b)**^			*P* < 0.01
Adenocarcinoma	2593(91.11)	7334(64.25)	
Signet ring cell	244(8.57)	1766(15.47)	
Other	9(0.32)	2314(20.27)	
**Grade** ^**c)**^			*P* < 0.01
Grade I	44(1.55)	1340(11.74)	
Grade II	1579(55.48)	3163(27.71)	
Grade III and IV	1223(42.97)	6911(60.55)	
**T term**			*P* < 0.01
T1	278(9.77)	2987(26.17)	
T2	351(12.33)	1675(14.67)	
T3	747(26.25)	4109(36.00)	
T4	1470(51.65)	2643(23.16)	
**N term**			*P* < 0.01
N0	903(31.73)	5295(46.39)	
N1	545(19.15)	2900(25.41)	
N2	567(19.92)	1509(13.22)	
N3	831(29.20)	1710(14.98)	
**M term**			*P* < 0.01
M1	358(12.58)	2012(17.63)	
M0	2488(87.42)	9402(82.37)	
**Race**			
White		1538(13.47)	*P* < 0.01
Black		7729(67.72)	
Other	2846(100)	2147(18.81)	
**Chemotherapy**			*P* < 0.01
Yes	2146(75.4)	6355(55.68)	
No	700(24.6)	5059(44.32)	
**Radiation**			*P* < 0.01
Yes	103(3.62)	3918(34.33)	
No	2743(96.38)	7496(65.67)	

Values are presented as mean ± SD or number (%). SD, standard deviation; SEER, Surveillance, Epidemiology, and End Results. (a) Location: Cardia: C16.0-Cardia; Fundus: C16.1-Fundus of stomach; Body: C16.2-Body of stomach. Gastric: C16.3-Gastric antrum; Pylorus: C16.4-Pylorus; Lesser curvature: C16.5-Lesser curvature of stomach; Greater curvature: C16.6-Greater curvature of stomach. Overlapping: C16.8-Overlapping lesion of stomach; Stomach: C16.9-Stomach. (b) Histology: Adenocarcinoma, 88,140/3: Adenocarcinoma; Signet ring cell carcinoma, 8490/3: Signet ring cell carcinoma. (c) Grade: Grade I, Well differentiated; Grade II, Moderately differentiated; Grade III, Poorly differentiated; Grade IV, Undifferentiated; anaplastic

### Patient prognosis analysis

**Fig. 1:**
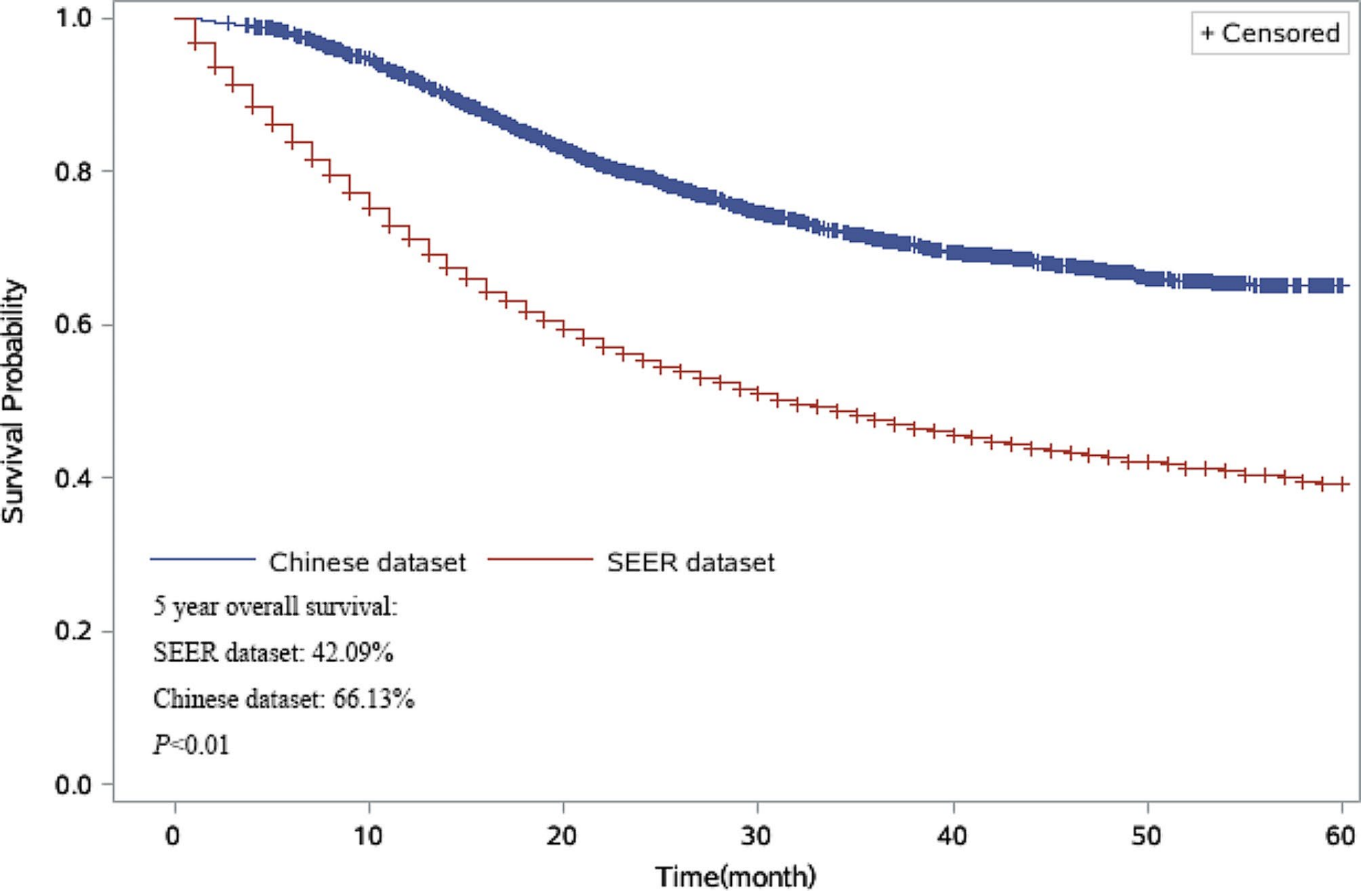
Comparison of the 5-year overall survival of patients in the SEER and Chinese datasets. SEER: Surveillance, Epidemiology, and End Results

**Fig. 2:**
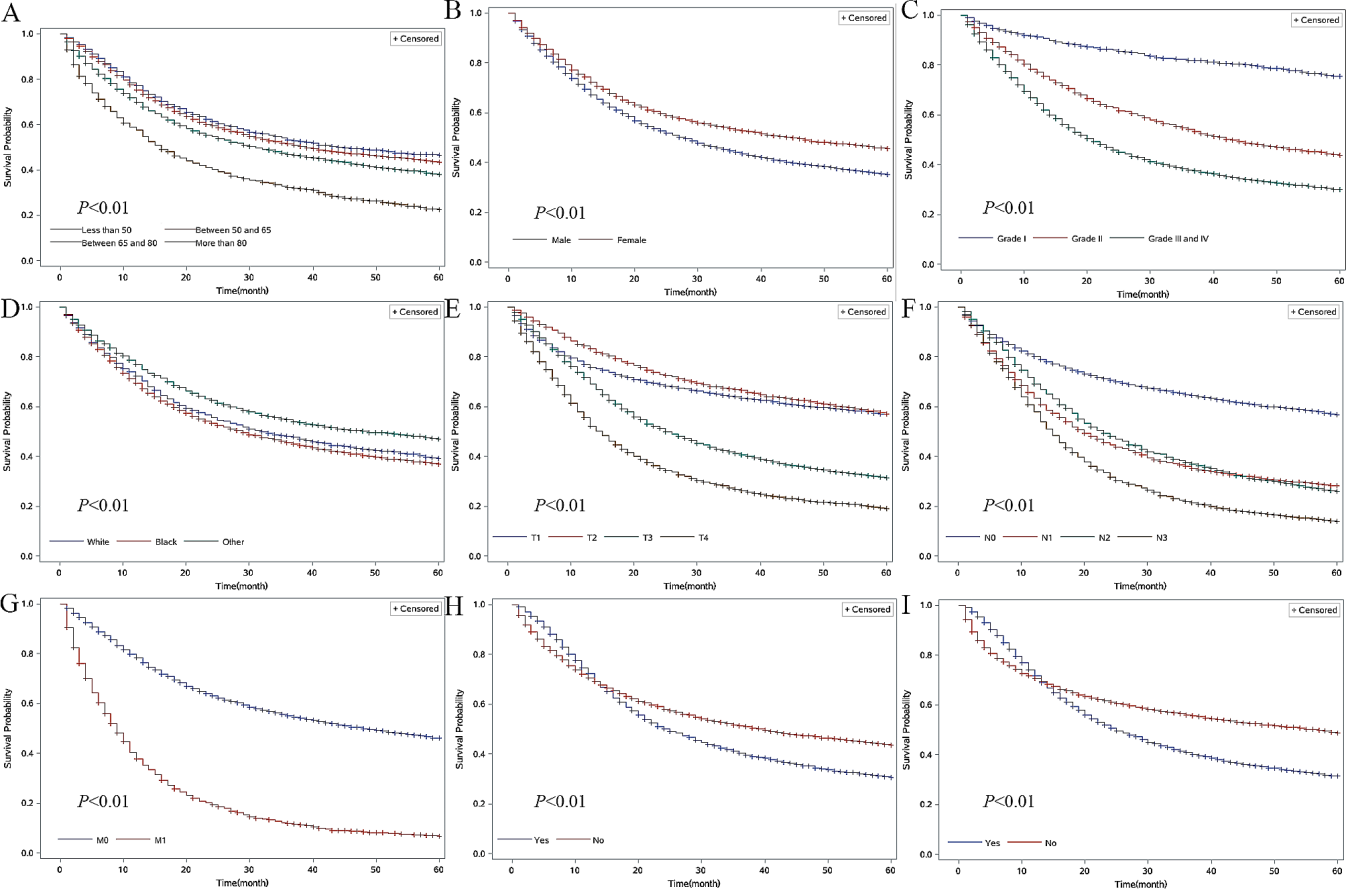
Kaplan–Meier survival analyses for overall survival of stomach cancer patients in the SEER dataset. (**A**): Comparison of the 5-year overall survival according to sex. (**B**): Comparison of the 5-year overall survival according to age. (**C**): Comparison of the 5-year overall survival according to different grades. (**D**): Comparison of the 5-year overall survival among patients of different races. (**E**): Comparison of the 5-year overall survival according to different T stages. (**F**): Comparison of the 5-year overall survival according to different N stage. (**G**): Comparison of the 5-year overall survival according to different M stage. (**H**): Comparison of the 5-year overall survival among patients undergoing radiotherapy. (**I**): Comparison of the 5-year overall survival among patients undergoing chemotherapy. SEER: Surveillance, Epidemiology, and End Results

**Fig. 3:**
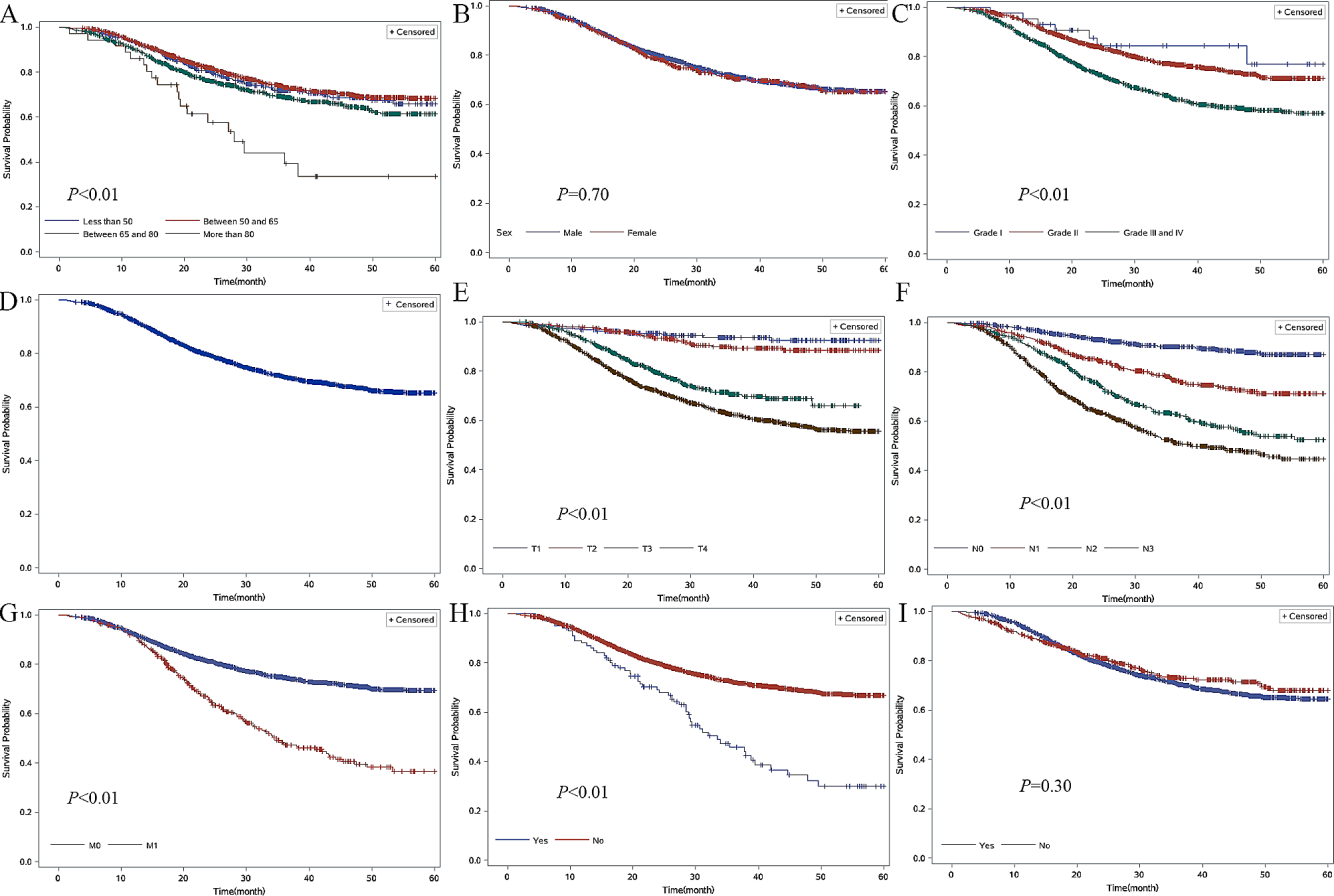
Kaplan–Meier survival analyses for overall survival of stomach cancer patients in the Chinese dataset. (**A**): Comparison of the 5-year overall survival according to sex. (**B**): Comparison of the 5-year overall survival according to age. (**C**): Comparison of the 5-year overall survival according to different grades. (**D**): Comparison of the 5-year overall survival among patients of different races. (**E**): Comparison of the 5-year overall survival according to different T stages. (**F**): Comparison of the 5-year overall survival according to different N stage. (**G**): Comparison of the 5-year overall survival according to different M stage. (**H**): Comparison of the 5-year overall survival among patients undergoing radiotherapy. (**I**): Comparison of the 5-year overall survival among patients undergoing chemotherapy

We conducted univariate analyses of overall survival in patients from the datasets to evaluate the impact of all variables on GC prognosis (Fig. 1). The results of the univariate analyses of overall survival indicate that poor prognosis was associated with age (patients aged 60–69 years, 70–79 years, and over 80 years, compared to patients younger than 60 years), pathological type (signet-ring cell carcinoma compared to others), grade (grades II, III, IV compared to grade I), and TNM stage in both the SEER and Chinese dataset. There was a significant association between patients with different tumor sites. In the SEER database, patients with tumors in the greater curve (C16.6-Greater curvature of stomach) were associated the poorest prognosis. However, in the Chinese dataset, patients with tumors in the stomach (C16.9-Stomach) were associated with the poorest prognosis (Figs. 2 and 3).

### Performance of ML method

**Fig. 4:**
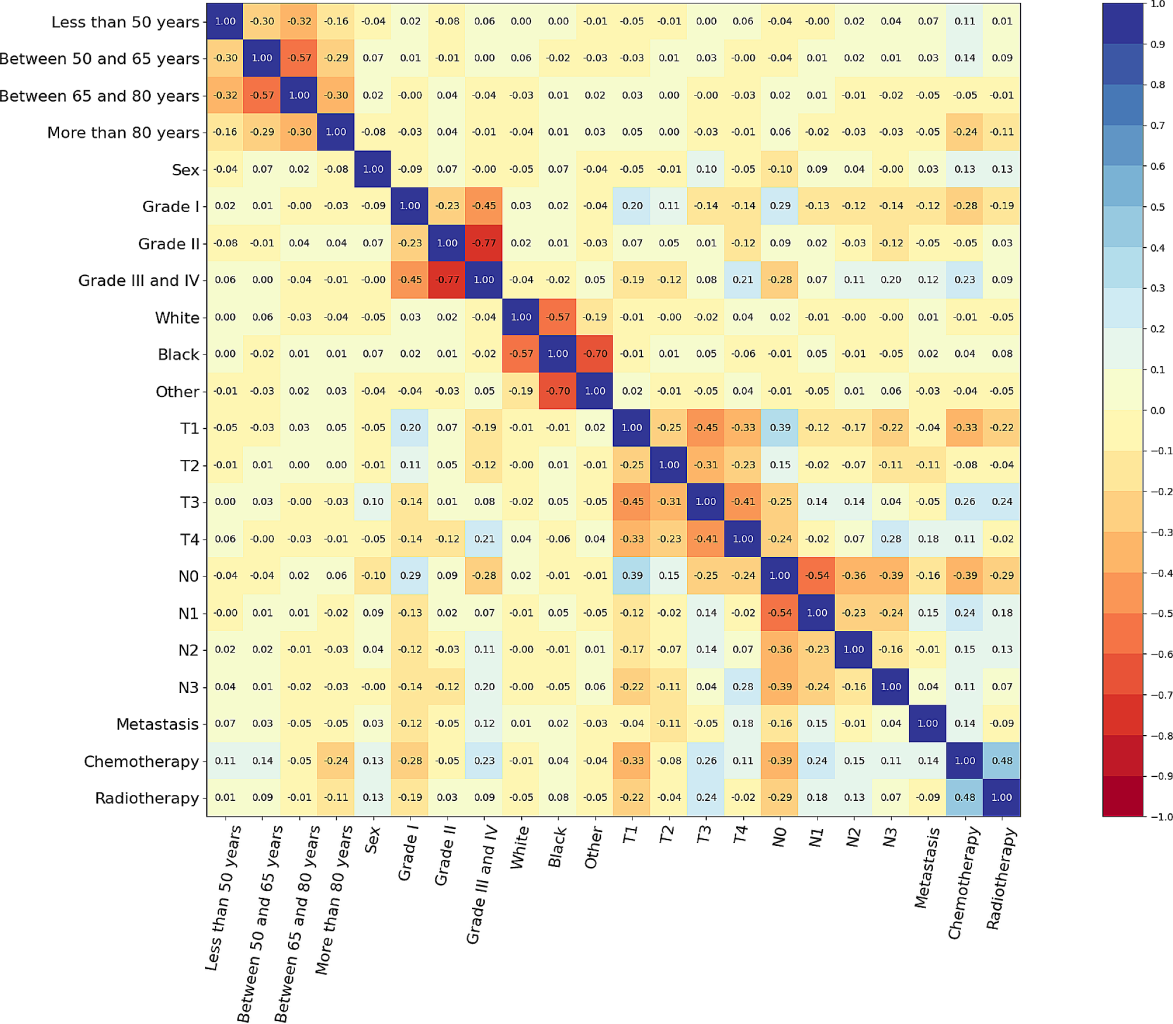
Correlation matrix of the variables for patients with GC. GC: gastric cancer

Correlation matrix showed a cluster of interconnected variables among the patients with GC (Fig. 4).

**Fig. 5:**
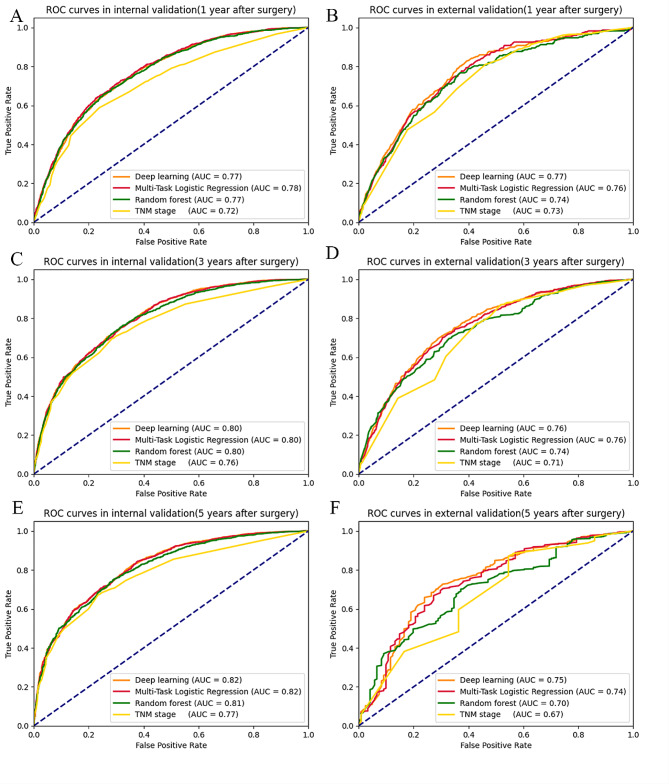
Comparison of the receiver operating characteristic curve using machine learning method and TNM term in internal and external validation. (**A**) 1 year after surgery in the SEER dataset (**B**) and 1 year after surgery in the Chinese dataset; (**C**) 3 years after surgery in the SEER dataset (**D**) and 3 years after surgery in the Chinese dataset; (**E**) 5 years after surgery in the SEER dataset (**F**) and 5 years after surgery in the Chinese dataset. AUC, area under the curve, SEER: Surveillance, Epidemiology, and End Results

The ROC curves in Fig. 5 demonstrate the performance of our ML models on the validation and external testing sets, compared to TNM staging. Our model showed consistent performance between the validation and external testing sets, and outperformed TNM staging in predicting overall survival with a higher AUC value. Deep learning was found to have the highest AUC value 1, 3, and 5 years after surgery (from 0.75 to 0.82). Multitask logistic regression and random forest also showed a good AUC for predicting mortality (from 0.74 to 0.82). The random forest algorithm showed a relatively poorer predicting capability (only 0.70) compared to the other two models when predicting the prognosis in external validation 5 years after surgery (0.74 and 0.75, respectively).

**Fig. 6:**
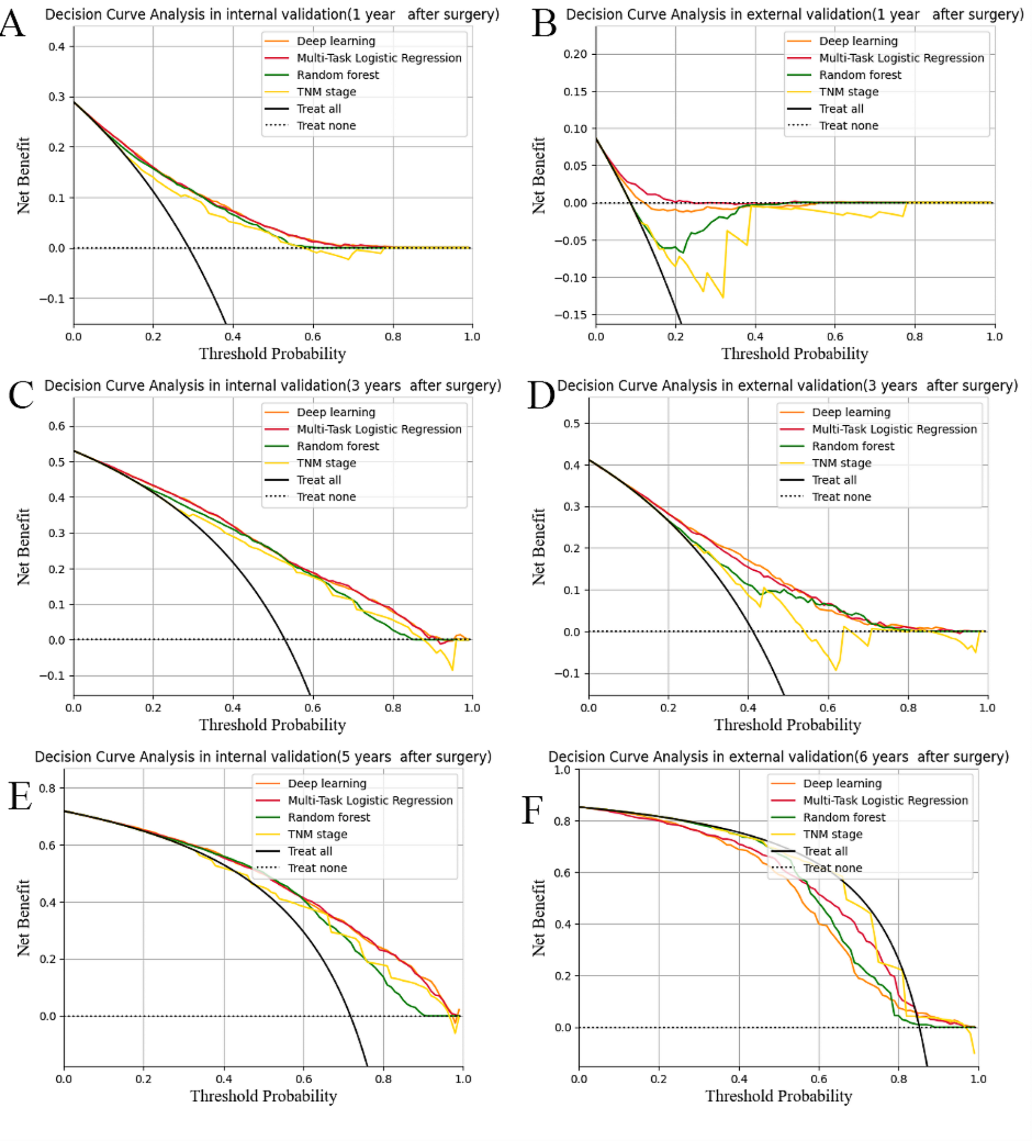
The decision curve for predicting patient survival. The clinical net benefit for each prediction model is calculated across a range of risk threshold probabilities. Clinical net benefit is defined as the minimum probability of disease at which further intervention would be warranted. (**A**) 1 year after surgery in the SEER dataset (**B**) and 1 year after surgery in the Chinese dataset; (**C**) 3 years after surgery in the SEER dataset (**D**) and 3 years after surgery in the Chinese dataset; (**E**) 5 years after surgery in the SEER dataset (**F**) and 5 years after surgery in the Chinese dataset. SEER: Surveillance, Epidemiology, and End Results

We next evaluated the performance of the models using decision curve analysis, to account for the impact on treatment decision making (e.g. surveillance vs. radical treatment). The heterogeneous profile of the patient population renders a uniform treatment strategy (treat all or no patients) inferior to strategies informed by any one of the four models (Fig. 6). When performing internal validation of the models, we found that TNM stage provided the least benefit compared to the other models, while deep learning yielded the highest net benefit. However, the gain from the models was more than zero only at 3 years in the external validation. The gain from TNM stage was much less than the other three models and the difference is even greater when compared with deep learning model.

**Fig. 7:**
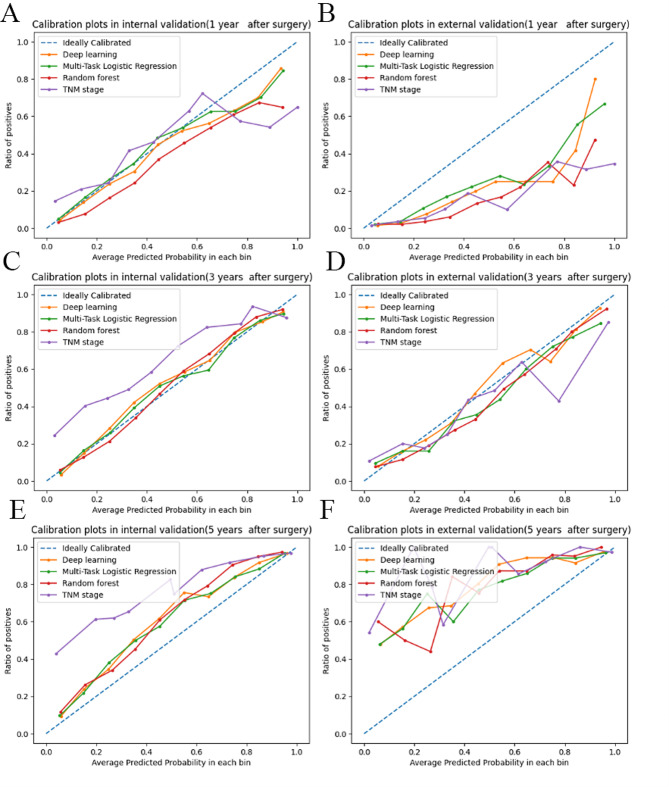
Calibration curves for predicting patient survival. Calibration curves for 1-, 3-, and 5-year overall survival in the SEER and Chinese datasets. By plotting overall survival on x-axis and the actual observed overall survival on y-axis, the calibration of the model can be evaluated. A line closer to 45 degrees indicates better calibration, suggesting that the predicted probabilities closely match actual outcomes. (**A**) 1 year after surgery in the SEER dataset (**B**) and 1 year after surgery in the Chinese dataset; (**C**) 3 years after surgery in the SEER dataset (**D**) and 3 years after surgery in the Chinese dataset; (**E**) 5 years after surgery in the SEER dataset (**F**) and 5 years after surgery in the Chinese dataset. SEER: Surveillance, Epidemiology, and End Results

The calibration plots for 1-, 3-, and 5-year survival in the internal validation cohort demonstrated favorable consistency between the ML prediction and actual observation (Fig. 7). However, only the calibration plots at 3 years showed favorable consistency in the external validation between the ML prediction and actual observation.

## Discussion

The current authoritative AJCC stage for predicting patient prognosis is hindered by widely varying clinical outcomes, despite similar staging and treatment regimens, suggesting that AJCC staging does not provide sufficient prognostic information [19, 20]. ML models represent a new approach that can improve prognosis prediction. Due to the autonomy-driven nature of ML, its ability to continuously add data as the patient progresses through treatment and visualize the prognosis of patients after GC is irreplaceable by all current predictive models [21]. Better prediction of prognosis, especially in the context of limited healthcare resources, allows us to identify patients with possible recurrence early and thus prevent them effectively, and has an impact on key clinical outcomes (e.g. mortality) [8, 22, 23]. Previous studies have identified prediction models and algorithms that surpass classical models such as the AJCC TNM staging system [24, 25]. In this study, we developed and tested ML-trained prognostic models for predicting 5-year postoperative GC survival. In contrast to the TNM stage prediction model, our ML-based models used 11 clinical features, including the AJCC TNM stage, to achieve optimal modeling. Their inherent data-driven capability allows multiple variables to be automatically combined to obtain high a AUC value and good calibration from previously undetected factors.

DL-based models, compared to traditional machine learning models including multitask logistic regression and random forest models, can more comprehensively reveal the underlying nonlinear relationships in data and handle more complex neural network models [9, 12]. A previous study constructed ML models, including the Boruta algorithm, neural network, support vector machine, and random forest, to accurately assess the overall survival of patients after GC surgery; however, no deep learning model was established for validation and comparison [26]. Our study introduced the DL-based model to predict survival rates and showed that the DL model achieved better performance compared to that of the AJCC TNM stage system and traditional ML-based models in individualized estimation of survival in GC patients. In recent years, DL models have successfully been applied for analyzing clinical, imaging, and genetic data [12–15]. However, the application of DL in predicting postoperative prognosis for GC patients has been limited. Zeng et al. developed a DL algorithm to predict survival rates for GC patients. However, the DL model was validated only using the SEER database and lacked validation with real-world data [17, 27]. In this study, a total of 11,414 patients with GC from the SEER database were included for internal validation and randomly divided into the training and testing groups at a 3:2 ratio. In addition, 2,846 GC patients from Henan Cancer Hospital were included for external validation to develop and test ML-trained prognostic models. Our study showed that DL-based models yielded more reliable predictions of survival compared to those by traditional ML and the AJCC stage models. These findings indicate the potential of DL models to significantly enhance individualized survival estimation for GC patients.

In our study, we found that the net gains of each model decision curve at 1, 3, and 5 years in the training set are larger than that in the test cohort, with multi-task logistic regression and DL slightly higher than TNM staging. In the test set, the net gain of each model was lower at 1 and 5 years, while at 3 years, the DL and multi-task logistic regression models were much more effective than the TNM staging. Moreover, the calibration curves showed that the training model fit was better than the test model for each year. In the test set, the accuracy of 3-year survival prediction was higher than that of 1-, 5-year survival prediction. The 1-year survival prediction probability was higher than the actual probability, and the 5-year survival prediction probability was lower than the actual probability. There are several reasons for this phenomenon: (1) the mortality rate of tumor patients is relatively low at 1 year and relatively high at 5 years, which makes the model identification more difficult. (2) The high accuracy of the training set and the low accuracy of the validation set may be due to the differences in the genetic background, race, and treatment of patients in the training set and the validation set. Methods such as transfer learning can be considered in subsequent studies to increase the accuracy in the validation set [28].

A limitation of this analysis is that the clinical prognostic model we developed does not incorporate any previously identified molecular markers that potentially influence patient prognosis [29–31]. Including these markers in our predictive model will enhance its reliability. Moreover, this was a retrospective study with some selection bias. A large prospective study is needed to validate the developed model to obtain more generalized and robust predictive validity of DL. Lastly, The algorithms we selected indeed established methods within the field of machine learning and have been extensively applied across diverse datasets in various domains. However, our intention in utilizing these well-established methodologies was to harness their proven strengths in handling complex, high-dimensional survival data, particularly in assessing their efficacy within a survival analysis context mirroring real-world clinical scenarios. The novelty of our work lies not in the introduction of new algorithms but in the comprehensive application and comparative analysis of these methods against the traditional TNM staging system within the specific domain of survival analysis.

The DL-based model constructed in this study achieves the first validation of the SEER database combined with the Chinese population database on internal and external data, which can more accurately assess the prognosis of postoperative GC patients and helps to provide accurate and personalized treatment for postoperative GC patients in a clinical setting.

## Conclusions

We constructed a DL-based model using data from both the SEER and Chinese population databases for the first time and subjected it to internal and external validation to predict the prognosis of postoperative GC patients. Our findings suggest that the DL-based model accurately predicts the survival rate of postoperative patients with GC.

### Electronic supplementary material

Below is the link to the electronic supplementary material.

Supplementary Material 1

## Data Availability

No datasets were generated oranalysed during the current study.

## Abbreviations

GC: Gastric cancer
AJCC: American Joint Committee on Cancer
ML: Machine learning
DL: Deep learning
SEER: The Surveillance, Epidemiology and End Results
LN: Lymph nodes
AUC: Area under the Curve

## References

[1] Sung H, Ferlay J, Siegel RL, Laversanne M, Soerjomataram I, Jemal A (2021). Global Cancer statistics 2020: GLOBOCAN estimates of incidence and Mortality Worldwide for 36 cancers in 185 countries. Cancer J Clin.

[2] Deng JY, Liang H (2014). Clinical significance of lymph node metastasis in gastric cancer. World J Gastroenterol.

[3] Yamada S, Hatta W, Shimosegawa T, Takizawa K, Oyama T, Kawata N (2019). Different risk factors between early and late cancer recurrences in patients without additional surgery after noncurative endoscopic submucosal dissection for early gastric cancer. Gastrointest Endosc.

[4] Pernot S, Voron T, Perkins G, Lagorce-Pages C, Berger A, Taieb J. Signet-ring cell carcinoma of the stomach: impact on prognosis and specific therapeutic challenge. World J Gastroenterol. 2015;21(40):11428–38. 10.3748/wjg.v21.i40.11428. 10.3748/wjg.v21.i40.11428 PMC4616218 26523107

[5] Kim YJ, Chung WC, Cho IH, Kim J, Kim S (2019). Prognostic effect of different etiologies in patients with gastric cardia cancer. Medicine.

[6] Eusebi LH, Telese A, Marasco G, Bazzoli F, Zagari RM (2020). Gastric cancer prevention strategies: a global perspective. J Gastroenterol Hepatol.

[7] Jiang Y, Jin C, Yu H, Wu J, Chen C, Yuan Q (2021). Development and validation of a Deep Learning CT Signature to Predict Survival and Chemotherapy Benefit in Gastric Cancer: a Multicenter, Retrospective Study. Ann Surg.

[8] Tran KA, Kondrashova O, Bradley A, Williams ED, Pearson JV, Waddell N (2021). Deep learning in cancer diagnosis, prognosis and treatment selection. Genome Med.

[9] Lee C, Light A, Alaa A, Thurtle D, van der Schaar M, Gnanapragasam VJ (2021). Application of a novel machine learning framework for predicting non-metastatic prostate cancer-specific mortality in men using the Surveillance, Epidemiology, and end results (SEER) database. Lancet Digit Health.

[10] Han JH, Lee S, Lee B, Baek OK, Washington SL 3rd, Herlemann A, et al. Explainable ML models for a deeper insight on treatment decision for localized prostate cancer. Sci Rep. 2023;13(1):11532. 10.1038/s41598-023-38162-1. 10.1038/s41598-023-38162-1 PMC10352331 37460568

[11] Brieu N, Gavriel CG, Nearchou IP, Harrison DJ, Schmidt G, Caie PD (2019). Automated tumour budding quantification by machine learning augments TNM staging in muscle-invasive bladder cancer prognosis. Sci Rep.

[12] Issa NT, Stathias V, Schürer S, Dakshanamurthy S (2021). Machine and deep learning approaches for cancer drug repurposing. Sem Cancer Biol.

[13] Daneshjou R, He B, Ouyang D, Zou JY (2021). How to evaluate deep learning for cancer diagnostics - factors and recommendations. Biochim et Biophys acta Reviews cancer.

[14] Kuntz S, Krieghoff-Henning E, Kather JN, Jutzi T, Höhn J, Kiehl L (2021). Gastrointestinal cancer classification and prognostication from histology using deep learning: systematic review. Eur J Cancer.

[15] Avanzo M, Wei L, Stancanello J, Vallières M, Rao A, Morin O (2020). Machine and deep learning methods for radiomics. Med Phys.

[16] Duggan MA, Anderson WF, Altekruse S, Penberthy L, Sherman ME (2016). The Surveillance, Epidemiology, and end results (SEER) Program and Pathology: toward strengthening the critical relationship. Am J Surg Pathol.

[17] Steyerberg EW, Harrell FE Jr. Prediction models need appropriate internal, internal-external, and external validation. J Clin Epidemiol. 2016;69:245–7. 10.1016/j.jclinepi.2015.04.005. 10.1016/j.jclinepi.2015.04.005 PMC5578404 25981519

[18] Heagerty PJ, Zheng Y. Survival model predictive accuracy and ROC curves. Biometrics. 2005;61(1):92–105. 10.1111/j.0006-341X.2005.030814.x. 10.1111/j.0006-341X.2005.030814.x 15737082

[19] Tan P, Yeoh KG (2015). Genetics and Molecular Pathogenesis of gastric adenocarcinoma. Gastroenterology.

[20] Sasako M, Inoue M, Lin JT, Khor C, Yang HK, Ohtsu A. Gastric Cancer Working Group report. Jpn J Clin Oncol. 2010;40(Suppl 1):i28–37. 10.1093/jjco/hyq124. 10.1093/jjco/hyq124 20870917

[21] Kaur I, Doja MN, Ahmad T (2022). Data mining and machine learning in cancer survival research: an overview and future recommendations. J Biomed Inform.

[22] Greener JG, Kandathil SM, Moffat L, Jones DT (2022). A guide to machine learning for biologists. Nat Rev Mol Cell Biol.

[23] Schmidhuber J (2015). Deep learning in neural networks: an overview. Neural Networks: Official J Int Neural Netw Soc.

[24] Song X, Liu X, Liu F, Wang C (2021). Comparison of machine learning and logistic regression models in predicting acute kidney injury: a systematic review and meta-analysis. Int J Med Informatics.

[25] Kourou K, Exarchos TP, Exarchos KP, Karamouzis MV, Fotiadis DI (2015). Machine learning applications in cancer prognosis and prediction. Comput Struct Biotechnol J.

[26] Liu D, Wang X, Li L, Jiang Q, Li X, Liu M (2022). Machine learning-based model for the prognosis of postoperative gastric Cancer. Cancer Manage Res.

[27] Zeng J, Li K, Cao F, Zheng Y (2023). Development and validation of survival prediction model for gastric adenocarcinoma patients using deep learning: a SEER-based study. Front Oncol.

[28] Alrowais F, S SA, Marzouk R, A SS, Rizwanullah M, Zamani AS, et al. Manta Ray Foraging Optimization Transfer learning-based gastric Cancer diagnosis and classification on endoscopic images. Cancers. 2022;14(22). 10.3390/cancers14225661. 10.3390/cancers14225661 PMC9688577 36428752

[29] Cristescu R, Lee J, Nebozhyn M, Kim KM, Ting JC, Wong SS (2015). Molecular analysis of gastric cancer identifies subtypes associated with distinct clinical outcomes. Nat Med.

[30] Bang YJ, Van Cutsem E, Feyereislova A, Chung HC, Shen L, Sawaki A (2010). Trastuzumab in combination with chemotherapy versus chemotherapy alone for treatment of HER2-positive advanced gastric or gastro-oesophageal junction cancer (ToGA): a phase 3, open-label, randomised controlled trial. Lancet.

[31] Mathiak M, Warneke VS, Behrens HM, Haag J, Böger C, Krüger S (2017). Clinicopathologic characteristics of microsatellite instable gastric carcinomas revisited: Urgent need for standardization. Appl Immunohistochem Mol Morphology: AIMM.

