# Supplementary material for: Development and validation of a deep learning model for predicting postoperative survival of patients with gastric cancer
Source: BMC Public Health. 2024 Mar 6;24:723. doi: 10.1186/s12889-024-18221-6 (PMC10916254; doi:10.1186/s12889-024-18221-6)
Supplement: Supplementary file 1 — Supplementary Material 1 [file 12889_2024_18221_MOESM1_ESM.docx]

**Additional File 1: Parameters of Machine Learning Models**

1. **Deep Learning (DL)**

activation: BentIdentity

num_units: 150

lr: 1e-3

init_method: xav_uniform

1. **Multi-Task Logistic Regression (MTLR)**

bins: 50

lr: 1e-3

init_method: orthogonal

1. **Random Survival Forests (RSF)**

num_trees: 200

max_features: sqrt

max_depth: 5

min_node_size: 20

alpha: 0.05

minprop: 0.1
